# Marine conservation in Vanuatu: Local conceptualisation and ‘assemblage’

**DOI:** 10.1007/s13280-022-01767-3

**Published:** 2022-08-27

**Authors:** Arno Pascht

**Affiliations:** Institut für Ethnologie, Ludwig-Maximilians-Universität München, Oettingenstr. 67, 80538 Munich, Germany

**Keywords:** Human–environment relations, Local knowledge, Marine conservation, Ontological differences, Oceania, Vanuatu

## Abstract

This article deals with the local conceptualisation of ‘conservation’ in the village Siviri in Vanuatu where villagers have established and maintain a small marine conservation area. Looking at villagers’ motivations, the aim is to carve out the local conceptualisation and practice of ‘conservation’, to show what conservation is for the villagers. The theoretical framework is a combination of two approaches, namely ‘assemblage’ and ‘world-making’. Conservation in Siviri is ontologically different from the concept of conservation used in Vanuatu national policy. It can be regarded as a creative engagement of villagers with their environment(s) to preserve the specific world-making assemblage consisting of humans and marine life for future generations.

## Introduction

When there is a conservation area, there will be many fish […] for the children in the future.^1^

(Female inhabitant of Siviri, Vanuatu)

Social science literature on conservation endeavours around the world often deal with misunderstandings between implementing organisations or Western scientists, on the one side, and participating local populations on the other (e.g. Blaser 2009, 2013; Homewood 2017). Scholars criticise the approaches of conservation organisations (Chua et al. 2020) as ignorant of local concepts and practices. This critique refers to top-down approaches of the organisations, which often cause conflict and miss the intended goals, including not being sustainable in the long term (e.g. Adams 2017; Homewood 2017; Howell 2017). In his argument for ‘conservation from below’, Bill Adams emphasises the need to extend the current formal definitions of ‘conservation’ which organisations use in their project design and implementations (Adams 2017, p. 119). He states that local definitions of conservation, including local ideas and daily practices, should be taken seriously and the “future of non-human biodiversity depends on the possibility of re-imagining conservation itself” (ibid. 2017, p. 121). People should be able to define what conservation is and can be, and accordingly determine the conceptualisation and implementation of projects which take place in their locality. This article deals with such definitions of what conservation is and can be for people in Vanuatu.

In Vanuatu, an island state in the South Pacific, population increase since independence in 1980 has led to increasing concerns about overfishing (David 1989). Since the 1990s, the national government has made efforts to introduce community-based fisheries management and marine conservation and since then, the concept and implementation of marine conservation areas has spread over the whole country. Subsequently, conservation, especially marine conservation at the community level, has become important in Vanuatu (Johannes 2002a, pp. 318–319; Bartlett et al. 2010; Raubani et al. 2017; Steenbergen et al. 2021).^2^ Ni-Vanuatu^3^ village communities have, in many cases, been the driving motor for the establishment and management of conservation areas (Hickey and Johannes 2002a).

Locally managed conservation areas also exist in other parts of Oceania (Johannes 2002a). In her article about comparable areas in the South Pacific state of Fiji, Elodie Fache emphasises two forms of value of reef ecosystems, as explicated by Foale et al. (2016): On the one hand, the economic (or utilitarian) value of such systems as a resource for securing livelihoods. On the other hand, an ‘intrinsic’ value to biodiversity, based on Western scientific models and the consideration of the accelerated extinction of species. She states that Melanesian populations do not generally share this latter value and the underlying epistemological premises (Fache 2020, p. 128). In a similar vein, Johannes (2002b) explicates that in some Pacific regions an “indigenous conservation ethic” exists, whereas in others not (Johannes 2002b, p. 6).

In Vanuatu’s conservation policy, biodiversity as well as utilitarian/economic values play a role, as shown, for example, by the National Sustainable Development Plan 2016 to 2030 (see below).^4^ Decentralised conservation practice with villagers as important players or even driving force has a 30-year tradition now, and the support of the people is mostly strong. Although authors of several studies mention a number of reasons for fast adoption of new marine resource management measures in Vanuatu (Hickey and Johannes 2002; Johannes 2002a), there are few mentions of the underlying motivations of villagers for establishing such measures. A few studies provide insights as to why people decide on conservation areas or marine protected areas (MPAs).^5^ Christopher Bartlett and his co-authors state that while “many of Vanuatu’s closures have conservation objectives, unstated secondary objectives abound. In contemporary practice, closures may represent an important avenue for development materials and aid, strengthened ownership and territorial claims, access to tourism, or political power” (Bartlett et al. 2010, p. 102). In a similar vein, Mark Love (2021) stresses that “MPAs can be an agentive appropriation for all sorts of reasons, e.g. legitimizing territorial claims, resource capture (attracting capital through tourism or donors), or an alternative means to impose sanctions” (Love 2021, p. 106) and concludes for one of his case studies that “Labo’s use of MPAs could be said to be a creative and strategic response to local particulars” (Love 2021, p. 106).

The ethnographic case I present here from my work with inhabitants in the village of Siviri on the main island of Efate in Vanuatu adds another example to the creative abilities of local actors, but also extends it to the wider world-making processes in which non-human actors are involved (see Tsing 2015). The conservation area in Siviri established by the community and currently maintained without the active involvement of an implementing organisation, confirms the creative abilities which Love (2021) refers to. I will show that here a main motivation goes beyond seeing fisheries a “source of organic nutritious animal protein” and a means to further “human development through income generating options” (Hickey 2008, p. 17). This does not mean that these do not play a role as ‘secondary’ objectives for Siviri villagers. My intention is, however, to show that their main motivation is as Bartlett et al. state, ‘conservation’, but that ‘Siviri conservation’ is not conservation as defined by Western science (see Smith and Wishie 2000).

I raise two questions: first, what was the main motivation for Siviri villagers to establish and maintain a locally managed conservation area?^6^ Second, what are the different motivations of the villagers and the government or NGOs for establishing and maintaining it?

Connected to these questions, the main aim of this article is to provide an interpretation of what conservation *is* for the people in Siviri—in contrast to the idea of conservation conceptualised by the government or NGOs. I will show that conservation in Siviri should be seen as world-making and maintaining: through their creative engagement with their environment(s), including fish and the sea, but also with measures and regulations of the nation state and NGOs, villagers intend to preserve existing relationships between humans and non-humans, and, generally, the assemblage which I call ‘Siviri marine management’.

## Theoretical framework

In order to answer the question what conservation is in my ethnographic example, I draw on the concept of ‘assemblage’^7^ and I refer to proponents of the ‘ontological turn’.^8^ Asking ontological questions like this means thinking through concepts (Henare et al. 2006), or better, conceptualisations and includes the possibility of a resulting notion of conservation which is altogether different.^9^

This question, namely what conservation (and sustainability) is, was also raised by Mario Blaser (2009). He concluded that ‘Yshiro conservation’ in Paraguay differs fundamentally from conservation as understood by governments, scientists and NGOs (Blaser 2009, p. 16).^10^ Members of parties participating in establishing a conservation programme did not recognise that “the conflicts that ensue from this particular kind of misunderstanding […] involve the continuous enactment, stabilization, and protection of different and symmetrically entangled ontologies or worlds” (Blaser 2009, p. 11). Considering the conceptualisation and practice of ‘conservation’ in Siviri, I assume in a similar vein the encounter of different ontological ways of making and being in the world.^11^

The theoretical framework I use here includes that I interpret ‘conservation’ in Siviri as one element of a wider assemblage which I call ‘Siviri marine management’. Anna Tsing emphasises the multispecies aspect, open-endedness and agency of the elements of an assemblage:

Assemblages are open-ended gatherings. They allow us to ask about communal effects without assuming them. They show us potential histories in the making. For my purposes, however, I need something other than organisms as the elements that gather. I need to see lifeways – and nonliving ways of being as well – coming together […] Assemblages don’t just gather lifeways; they make them. (Tsing 2015: 22–23)

Tsing uses assemblage as gathering of diverse “rhythms, as they result from world-making projects, human and not human” (Tsing 2015, p. 24) and states that patterns of “unintentional coordination develop in assemblages” (Tsing 2015, p. 23). Similarly, I use this concept to focus on the various elements of the assemblage ‘Siviri marine management’ as results of world-making projects (Tsing 2015, p. 24) and their interactions with conservation as one of these elements. These elements described below are not considered disparate by Siviri villagers. I want to highlight that it is reasonable to call this assemblage in Siviri a “multispecies assemblage” (Tsing 2015, p. 181)^12^ or a “heterogeneous assemblage”—“a lively assemblage of humans and more-than-humans” (Blaser 2014, p. 50).

Referring to Deleuze’s and Guattari’s seminal book (1987), Tanya Li (2007) has developed an approach of assemblage which she uses for community forest management, including conservation, in Indonesia and elsewhere. She highlights three aspects of this concept: first, practice—assemblage “links directly to a practice, to assemble” (Li 2007, p. 264), second, the “continuous work of pulling disparate elements together” (ibid.) using a particular set of practices related to government, and third, the agency by “recognizing the situated subjects who do the work of pulling together disparate elements” (Li 2007, p. 265). While I also focus on the practice, i.e. assembling, and on the agency of actors in the assemblage, I do not analyse in detail the practices related to government or the work of pulling disparate elements together. I also draw on the concept of ‘assemblage’ because it does not presuppose entities in an essentialist manner (DeLanda 2006, p. 252).

In this article, I additionally employ the idea of ‘world-making’. In order to include not only humans as actors, I borrow Tsing’s approach that “every organism makes worlds; humans have no special status” (Tsing 2015, p. 292, note 7). This is in line with other authors, who claim that animals or things have to be considered as active and constitutive participants in the world (making) (e.g. Kohn 2013).

By using the concept of ‘world’ or ‘world-making’, I emphasise that I do not deal with ‘worldviews’ as representations of the one world ‘out there’ (Henare et al. 2006). I am concerned with ontological questions (Holbraad and Pedersen 2017, pp. 5, 11) and ontological alterity (Holbraad 2009), with a focus on the everyday creation of worlds through the participation and interactions of human and non-human actors—all those actors create something new, a world or multiple worlds (Henare et al 2006, p. 13; Pedersen 2012). I assume the possibility of a plurality of worlds (Viveiros de Castro 1998, p. 469; Pascht 2022) which differ regarding definitions about what a world is and what its constituents are (Holbraad 2013).^13^ These worlds are not marked by clear boundaries but overlap, intersect and interact (Blaser and de la Cadena 2018). I will show how people in Siviri create or make their world which they conceptualise not as created from separate spheres of environment and sociality/culture but where those ‘spheres’ (which do not exist as separate entities for Siviri villagers) are variously intermingled (Hetzel and Pascht 2019).

## Methods

This article is based on long-term anthropological fieldwork. A part of the research was conducted over a period of 15 months between 2016 and 2019 for a project on human–environment relations and climate change.^14^ The other part took place in 2020 for the project SOCPacific^15^ where the focus was on marine conservation and fishing in the village of Siviri.^16^ The main methods used were participant observation, including practical participation in the various fishing activities of villagers, such as fishing with a net or diving. I also participated in festivities for which the conservation area was temporarily opened for one-off harvesting. Additionally, different forms of interviews and conversations were used—mainly semi-structured interviews (Kvale 2007) and freelisting (Quinlan 2005) about key concepts in connection with marine conservation with subsequent discussions. All interviews were conducted in Bislama.^17^ During these two research projects, approximately 120 formal interviews were conducted and numerous informal conversations, including during fishing trips or at the beach, with Siviri villagers of different gender and age. About the same number of interviews with men and women were conducted and it was also important to consider members of different categories and status: chiefs, representatives of the Presbyterian Church, men and women without chiefly rank, wage labourers, self-employed persons, casual labourers etc. The statements of members of these different status categories etc. were rather coherent: there were no fundamental differences regarding people’s relation to the ocean and to fishing/reef gleaning. A main gender difference was that women mainly went gleaning and line fishing at the beach, and sometimes fishing with a net, whereas men (young and old but not children) went spearfishing as well as fishing with a net, but did not collect shellfish. Additionally, interviews were conducted with a representative of the Nguna-Pele Marine and Land Protected Area, and staff of the fisheries department.

## Research site

The Pacific Island State of Vanuatu consists predominantly of islands of volcanic origin (Nunn 2003) which, including the main island of Efate, were uplifted from the ocean and further built up subaerially (Stewart et al. 2010, p. 314). Vanuatu, independent since 1980, is located 1750 kms east of Australia and 500 kms northeast of New Caledonia (Dumas and Fossey 2009). Efate is one of its largest islands, with the highest elevation of Mount MacDonald at 647 m (ibid.). Efate is complemented by offshore islands in the north, Nguna, Pele and Emau, which are also volcanic islands (ibid.; Stewart et al. 2010, p. 307). Efate has a fringing reef, which is “a few metres to tens of metres deep at most” (Stewart et al. 2010, p. 314). Most villages are located along the coast; some with direct, other with approximate, access to the sea. The ring road on Efate connects the village ‘narasaed’ (Bislama for ‘on the other side’), the northern part and the off-shore islands, with the capital of Vanuatu, Port Vila. Villagers in Vanuatu today usually live in extended families of several generations. Many of the ni-Vanuatu living on Efate commute from rural areas to Port Vila for wage labour, to sell garden yields in the markets, for education or for enjoying leisure time.

The village of Siviri is located on the north coast of Efate. It comprises approximately 200 inhabitants. Villagers are Christians and the predominant denomination is Presbyterian. The majority belongs to four related families who are the (customary) owners of the land and the lagoon of the village. The people of Siviri have a long tradition of combining horticulture and fishing. Today, villagers regard their gardens as important because they either supply food for daily life or income through selling crops at the market in Port Vila. Furthermore, they also provide important crops such as yam for ceremonial purposes. Villagers see fishing rather as an additional source for food or—more rarely—income, in contrast to root crops, which are regarded as the most important staple food besides rice, which is not cultivated in Vanuatu but is imported and can be bought in stores (Hetzel and Pascht 2019).

## National legal and policy background for marine conservation in Vanuatu

The Environmental Management and Conservation Act, which came into effect in 2003, shows the growing significance of conservation in Vanuatu.^18^ In this policy, a number of regulations for community conservation areas have been determined. For example, the possibility for customary landowners^19^ to register such an area is provided.

In 2016, the government of Vanuatu presented the ‘2030 National Sustainable Development Plan 2016 to 2030—the People’s Plan’.^20^ This plan refers to the Sustainable Development Goals of the United Nations, and specifically elaborates sustainable development’s three ‘pillars’: society, environment, economy. The description of the environment pillar states: “The environment pillar seeks to ensure a pristine natural environment on land and at sea that continues to serve our food, cultural, economic and ecological needs, and enhance resilience and adaptive capacity to climate change and natural disasters” (Department of Strategic Policy, Planning and Aid Coordination 2016, p. 13). This statement establishes a dichotomy between the environment (as pristine and natural) on the one hand, and humans who use this environment for their needs on the other.

In the Sustainable Development Plan, the issue of conservation is taken up under two sub-goals of the environment pillar, namely natural resource management and, second, ecosystems and biodiversity. The first explicitly mentions fishing when it demands the promotion of “sustainable development of the fisheries sector that values the protection and conservation of marine and freshwater resources” (Department of Strategic Policy, Planning and Aid Coordination 2016, p. 14).^21^ The second does not include a reference to resources, but highlights biodiversity with the following goal: “Protect biodiversity and ecosystems and their significant role in our culture, society and environment” (ibid.: 15). In the plan, thus, quite different assumptions about human–environment/nature relations are mentioned.

Both the Act cited above and this Sustainable Development Plan were drafted within the last 20 years. However, even before this, marine conservation, and especially community-based conservation, was known in Vanuatu: Robert Johannes states that of “27 villages surveyed in 1993, only 1 had not introduced MRM [marine resource management] measures in the previous four years”, i.e. measures “employed consciously to reduce or eliminate overfishing or other damaging human impacts on marine resources”. He stresses that enforcement “was by village authorities, not the Fisheries Department” (Johannes 2002a, p. 318). In a more recent publication (Bartlett et al. 2010) the authors also report community-established protected areas in Vanuatu and the wider Pacific islands region (Bartlett et al. 2010, p. 99). They draw attention to the multiple terms for those areas. Different legislation, they state, use different terminology (Bartlett et al. 2010, p. 100). Their result regarding this terminological variety is that “while protected area terminologies were originally introduced by the central government through national legislation, they are now widely and diversely applied throughout the archipelago by a variety of village stakeholders, NGOs, customary organizations and the national government” (Bartlett et al. 2010, p. 101).

Francis Hickey notices that in the early 2000s generally, regarding conservation approaches, “government policy makers and bureaucrats, often educated in industrialized countries and increasingly isolated from rural communities, often acquiesce to the introduction of Western models, following the locally entrenched notion that ‘the West knows best’” (Hickey 2006, p. 21; see also Hickey 2007, 2008). This trend is opposed to the practice of leaving the responsibilities and praxis of conservation to village communities as stated by Johannes (2002a, b). Christopher Bartlett and his co-authors emphasise that in “almost all Vanuatu cases, customary leaders retain control over closure duration, regulation and enforcement, regardless of the closure name or primary area objective. The village chief is most commonly responsible for the declaration and revocation of a natural resource closure, and although respect for the chiefly institution has been eroded throughout the region, it remains robust in most parts of the Vanuatu archipelago” (Bartlett et al. 2010, p. 101). Chiefs and village council in Siviri correspond to this pattern: they are widely respected and have power to sanction. In this village, the chiefs, as representatives of the village community, have established and the community has, until now, managed their own marine conservation area themselves. What are the villagers’ ideas behind this conservation area in Siviri? Do their ideas refer to or adopt the ideas mentioned in the Vanuatu Sustainable Development Plan and other regulations?

## Establishing and maintaining marine conservation in Siviri

The establishment of the Siviri conservation area must be seen in the context of the establishing of numerous other similar areas in Vanuatu (see Bartlett et al. 2010), especially the Nguna-Pele Marine Protected Area Network which is located on the small islands of Nguna and Pele very close to Siviri.^22^ When I first asked my host family in Siviri about the local marine conservation area, one of the features they mentioned was the existence of a committee in charge. This committee consists of a male chairperson, a male conservation monitor, a representative of the women of the village and a male representative of the youth—representation of different social categories of the community is a common structure of committees in Vanuatu. All are volunteers who have agreed to take on this position. Two members of this committee told us that in the 1990s, the Fisheries Department promoted the idea of conservation and inhabitants of the village attended workshops about conservation held by Wan Smolbag, a local NGO.^23^ They explained that the people of Siviri accordingly established a marine conservation area to protect a section of the lagoon of the village.

**Fig. 1:**
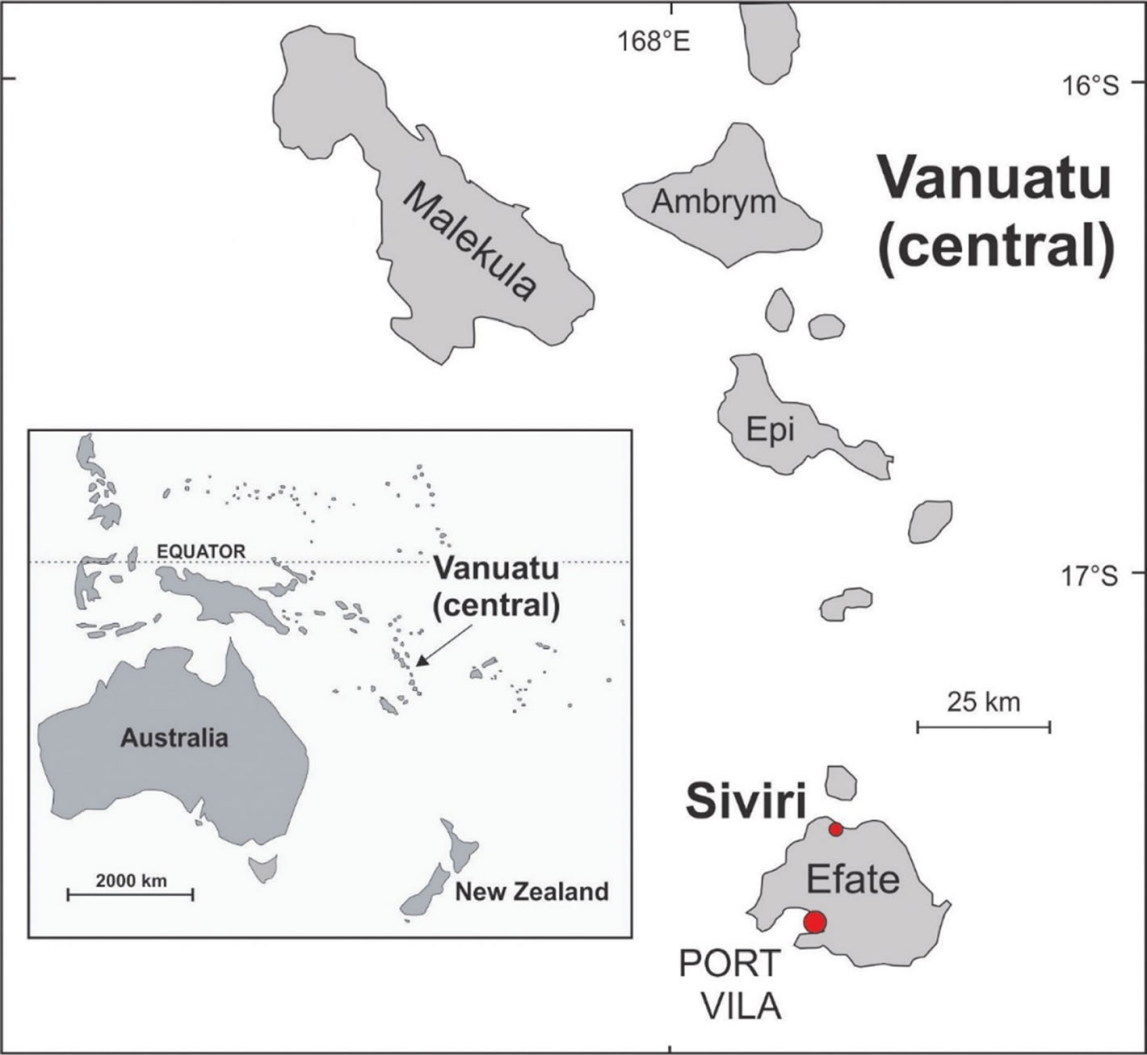
Map of Vanuatu (Patrick Nunn)

The first established conservation area was located quite a distance from the central village and members of the committee explained that it was not successful as a result of this and the associated difficulty of monitoring the area. According to them, there were numerous breaches of the ban on fishing and reef gleaning connected with the conservation area—mainly from people of neighbouring villages. At the beginning of the 2000s, the committee decided to relocate the conservation area closer to the centre of the village—namely to the lagoon directly in front of the main part of the village where most of the houses and the church are located—therefore visible to everyone living in the village. This conservation area remains in existence in the same location today. Villagers usually refer to it as ‘conservation area’ or just ‘conservation’.^24^ Its length measures about 700 m (see Fig. 1). There are no barriers, so everybody has access to this part of the lagoon and the associated sandy beach area is open to the public, including visitors for swimming and snorkelling.

**Fig. 2:**
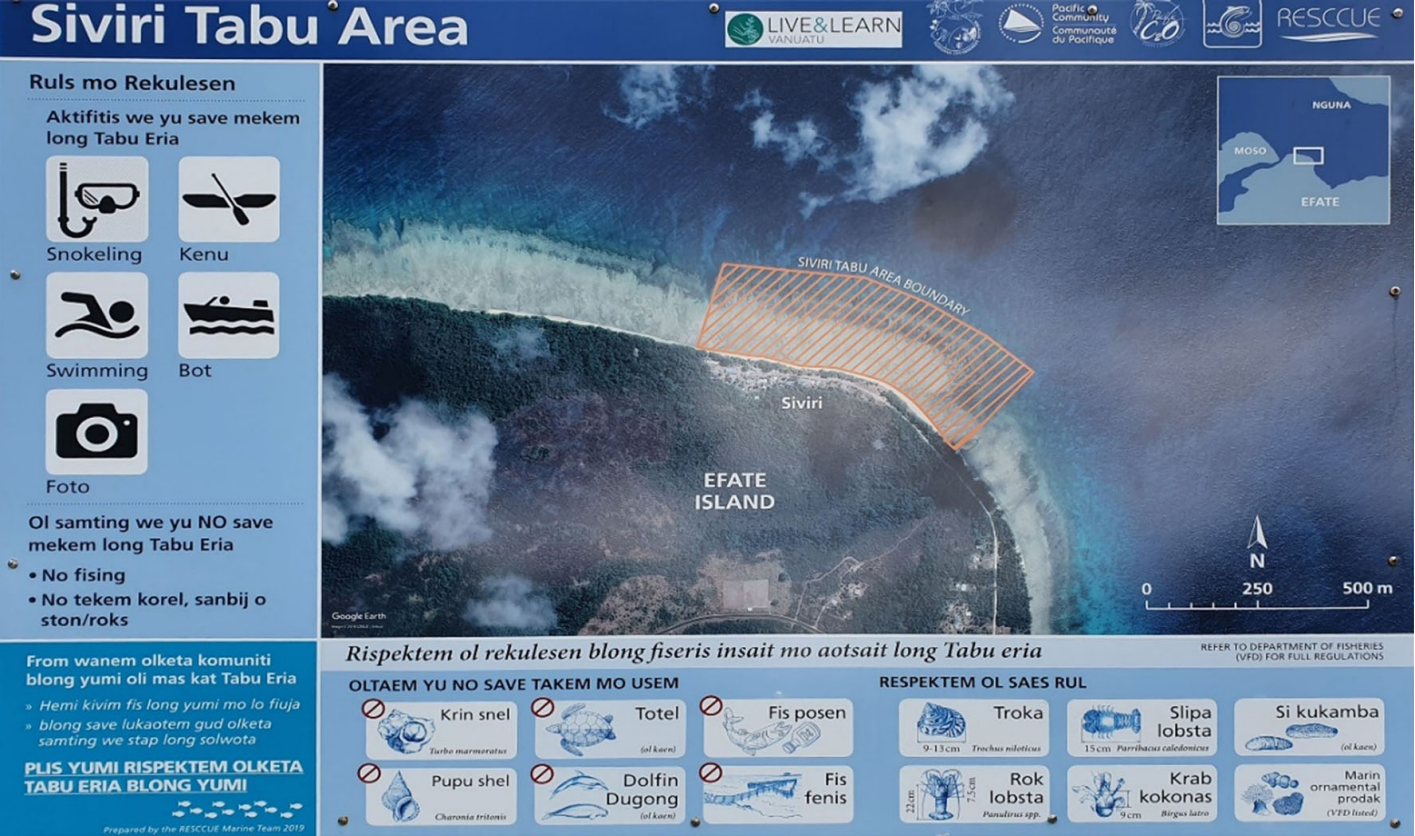
Sign-board for the Siviri Conservation Area

Without the villagers knowing beforehand, a cooperation of NGOs, environmental projects and regional organisations erected in 2020 a large sign-board for this conservation area. The area is named there as ‘tabu area’, an unusual expression in the village,^25^ and regulations as to what is allowed and what is forbidden are specified. In fact, the regulations referring to the conservation area as stated on the board do not wholly coincide with the regulations my interlocutors explained to me.

To date, the marine conservation area has been exclusively managed by the village community, represented by the conservation committee. My interlocutors explained that the regulations for this conservation area are simple: people are neither allowed to catch fish, nor to collect shellfish, whereas swimming, snorkelling or using boats for recreation or transport is allowed. Several times a year, the conservation area may be opened for fishing. According to my interlocutors, it had only been opened once for one day in 2020, Chief’s Day, an important national holiday in Vanuatu, celebrating the so-called traditional chiefly system and its representatives (Lindstrom 1997). Besides festivities and parades in the capital Port Vila, people in the villages gather on this day, celebrate and eat together. In Siviri, this is also the day when the first yam of the year is harvested, blessed in church and then other ceremonies follow. For this occasion, the conservation area is opened for fishing to complement the festive dishes.

The chairperson of the conservation committee remembered that the area was only opened twice in 2019, first, for the Chief’s Day, and second, for a gathering of church leaders which took place in Siviri. When the area is opened, every inhabitant is allowed to catch fish of large size only, but not permitted to gather shellfish.

Breaches of the rules of the conservation area result in sanctions. For 2019, the chairman told us that three people did not respect the rules and went fishing in the conservation area—all were Siviri villagers. In each case, there was a community meeting where the offenders were fined. The fine for the breaches was one woven mat and 5,000 Vatu (about 40 Euros).

## Marine life in Siviri: interactions

People in Siviri grow up watching fish, interacting with fish and eating fish.^26^ Children learn from an early age how to go fishing and how to collect shellfish and most of the adults practise it on a regular basis. While female interlocutors engage either in collecting shellfish on the reef or in fishing with a line, men frequently fish with different kinds of nets or dive (during the day or at night-time) and use spear guns to catch fish. Especially for young men, this is popular and the most skilled become well-known in the community. Joining Siviri villagers when they went fishing, I experienced their enjoyment of the activity of fishing or gleaning shellfish; they were proud of their catch, and they often emphasised how good and healthy it is to eat fresh fish. One inhabitant told me that he went spearfishing alone during the day, the fish being cooked by the extended family in the evening. A member of my host family and one or two neighbours regularly went spearfishing at night. Returning home, they cooked a part of the catch, ate it and drank together until late, while the rest of the fish was put in the freezer for the families—sometimes so many fish were caught that some were able to be sold. A couple who lived near my accommodation told me that they go fishing with their three children, mostly during school holidays, as a family enterprise, the fish being consumed by members of the extended family. Two other neighbours went and laid out a net, and at the next day I went with them to collect the fish. The catch then was shared among the families of the two. One day I went with my host family, including further relatives who do not live in the same household, for a picnic by boat. During the enterprise, various fishing and reef gleaning activities took place—the men went spearfishing; during the boat trip, they fished with a line; women, children and myself went on the reef and collected shellfish. Most of the fish and shellfish was consumed during the picnic and a small part was taken home afterwards. A female relative of my host family regularly gathers children of the village to collect shellfish which is then shared among the extended families of the children. A woman who lives in Siviri up the hill told me that she regularly goes alone on the reef to catch fish with a line for the extended family and another woman told me that she would visit the lagoon and the reef to catch octopus the next day.

The frequency of fishing activities of the villagers depends mainly on individual preferences. Some of my interlocutors told me that they fish every two or three days, whereas others indicated that they only go twice or three times per month. Fishing and collecting shellfish are popular leisure time activities.

As mentioned, fishing may also be used to generate monetary income. The most skilled fishermen are known for their skills around the island and take orders from people who then collect the catches for celebrations or family gatherings. Several of my interlocutors pointed out that fish also act as a tourist attraction because tourists sometimes visit Siviri to snorkel in the lagoon.

All my interlocutors explained that fish is a food that they enjoy having on their plates: “Fish is our food. I like to eat fish”. Most have a favourite fish whose taste they prefer. Fish is not only ‘food’, but is ‘healthy food’ and can even be used as medicine. My interlocutors never spoke of fish or shellfish as an important staple food but rather described it as a healthy supplement to other food like root crops or processed food like rice.

In contrast to fishing, which is not connected with a certain time structure in Siviri, gardening structures the social lives of ni-Vanuatu (Mondragón 2009). Root crops, especially yam, play an important role in Siviri and in fact in the whole of Vanuatu, not only for a daily food supply, but also for ceremonies like marriages (Hickey 2006, p. 17; Rio 2007). As opposed to the key role of root crops, interlocutors from Siviri stated unanimously that fish and other seafood does not play a role in ceremonies. On Chief’s Day, when most villagers fish in the conservation area, the catch is consumed by the inhabitants of Siviri during the festivities on this day, but fish is not used in the ceremonies itself, which focus rather on the first harvest of yam in the cultivation cycle. According to one interlocutor, there is a trend that fish is served as food for participants in other ceremonies, for example weddings.

Additionally, Siviri villagers emphasised that fish are actors themselves, maintaining the environment for themselves and humans. “Fish are cleaning the reef and the water,” several interlocutors stressed. A villager remarked that fish act not only as food for humans, but also for other fish. Interlocutors mentioned the aesthetic aspects of fish: fish, they explained, are very colourful, they “colour the reef”. “Fish are a good thing. They help people”. This statement summarises what Siviri interlocutors told me: they regard fish as very positive and they assume that they help people in the various ways mentioned by my interlocutors. As the statement of an interlocutor shows, fish have agency because they are able to move to the conservation area where they cannot be caught (see below).

With these short examples I want to show the manifold occasions, contexts and forms in which people in Siviri interact with marine life and that the various social and economic interactions among people are inevitably connected with these interactions which form an important part of the lifeworld of Siviri villagers. Marine life contributes to maintaining the (future) environment and the life of the villagers and are in various ways part of the way of living of the villagers and, in my interpretation, a part of their being in the world.

## ‘Siviri (marine) conservation’: Marine life and humans

Conservation is good to maintain a great number of fish and birds.

(Male inhabitant of Siviri, Vanuatu)

All our interlocutors emphasised that the marine conservation area is effective for maintaining or even increasing the number of fish and other non-humans—equivalent to terrestrial conservation, which increases the number of birds (and other animals). Why is this so important for the villagers that they gave up the possibility to fish and gather shellfish in an area which is easily accessible for them?

One of our interlocutors explained that with the establishment of the conservation area, Siviri villagers created a sanctuary for fish to enable them to hide and thus avoid being caught by villagers. “I want to catch them, but they know they can swim a few metres up and are safe”, one man laughed. Although this means that on some days, he would go home empty-handed, he acknowledged that ultimately, this benefits fish as well as people. The character of the conservation area as a sanctuary, interlocutors explained, is also advantageous for tourists visiting the village: the fish are particularly tame there and therefore easy to observe even for inexperienced snorkelers. “The fish know that they don’t have to worry about anything in the conservation area and are therefore quite relaxed”, explained a member of the conservation committee.

Siviri villagers explained that the conservation area would be of advantage to themselves, but also emphasised that it was important that their children in the future encounter the same realities as exist today—conservation means that the “next generation can still enjoy the same as you today” explained an inhabitant of Siviri in his thirties. Future generations will benefit from the possibility to obtain healthy food, knowing and living in the same environment as people of today. A woman phrased it a little differently: “when there is a conservation area, there will be many fish, trees and animals for the children in the future”. Furthermore, she explained that a conservation area also implies that people have respect for everything that is located inside. Interviews and freelistings with many other men and women of different ages in Siviri showed great similarity to these statements.

Striving for continuity by creating a conservation area in Siviri can be interpreted as analogous to striving for continuity regarding cultivation: fish and fishing are an important part of the present reality of the people—and of future life. Similar to the field of cultivation, where ni-Vanuatu are enthusiastic about learning and creative in introducing new methods (see Hetzel 2021), with the introduction of a conservation area, people created with a new concept the means to maintain the possibility of continuing with fishing in the future, and, more generally to maintain the relationships between people and fish.

As shown in the previous section about human–marine life interactions, one important aspect for people in Siviri is that fish are a resource, mainly for the provision of food or monetary income. Accordingly, people mention the role of conservation as to “take care of our resources for the benefit of future generations”. Many of our interlocutors gave very similar explanations and underlined the importance of preserving fish stock. However, fish, as also presented above, are not only a resource, they are also in other ways active parts of and participants in the environment because they can help people. As a result, they are actively involved in the creation of the environment or world in Siviri. Conservation is thus not only about preserving resources; it is also about preserving the multispecies world of the Siviri villagers.

In summary, when inhabitants of Siviri encountered the concept of conservation during workshops and awareness events, they adopted the term and created a new practice designed to meet their own needs, namely to establish a system which they assume is appropriate to secure the continued existence of the species of fish and shellfish which existed in the past and which exist in the present.

## Results and Discussion

In the following paragraphs, I point out how ‘Siviri conservation’ may be approached to make sense of ethnographic materials. I will then contrast this with ‘conservation’ as it is used by the state and other organisations.

In Siviri, there is no conflict regarding marine conservation because of a misunderstanding of concepts such as ‘conservation’ or ‘sustainability’ based on Western science and introduced by the state or by development organisations. No local understandings of conservation or sustainability are ignored (Homewood 2017) and no conflicts occur due to misunderstandings because of the encounter of different ontologies such as that found in Blaser’s depiction of Yshiro conservation (Blaser 2009). Additionally, and in contrast to the assemblage analysed by Li (2007), the Siviri marine management assemblage is not characterised by severe tensions,^27^ but by the collaborative work of the whole community of the village, especially the chiefs and the members of the conservation committee.

Although people in Siviri use the word ‘conservation’ to refer to their locally managed marine area, I came to a similar conclusion as Elodie Fache for Fiji (Fache 2020): Siviri conservation is not a hybridisation of customary marine tenure practices and conservation promoted and practised by the state and conservation organisations. In contrast to the Fiji case, however, it may be misleading to speak of a ‘living tradition’ (see Fache 2020) in the case of the current Siviri conservation area because people of Siviri did not relate this area to a ‘traditional’ prohibition to catch fish at certain periods of time as reported for many places in Oceania, including parts of Vanuatu (Hickey 2006). Instead, it is adequate to conclude that Siviri villagers have created a new practice as ‘ontological innovation’ (see Salmond 2017) when they encountered ontologically different ideas and practices of ‘conservation’ from various sources.

I argue that this innovation which I call ‘Siviri conservation’—the conservation committee, conservation rules, sanctions, etc.—is one of the elements and thus one of the world-making projects (Tsing 2015) of the local multispecies (or heterogeneous) assemblage of ‘Siviri marine management’, which consists furthermore of humans (the inhabitants of Siviri); fish and other marine life in the ocean close to Siviri; diverse practices of fishing, including social practices (techniques and social organisation of fishing, circulating/exchange, consumption); knowledge (about marine life), boats, ideas about the future; ideas and discourses about numbers of fish and diversity of marine life; monetary income. The statements of my interlocutors are consistent with results of studies in various parts of Oceania which conclude that interpreting concepts and practices on the basis of a Western nature-culture dichotomy is not adequate (Mondragón 2018, p. 37; Pascht 2019; see also Rudiak-Gould 2016, p. 263). Elements of the local Siviri assemblage, including marine life and human life, are entangled in various ways and are active parts of it.

In other words, Siviri conservation can be interpreted as ontological innovation of Siviri villagers to maintain their world, which at the same time is a new world, because it now includes the element of ‘conservation’. By introducing and continuing to practise conservation, they sustain this assemblage which consists of various world-making projects. Thus, Siviri conservation is a way for villagers to creatively secure the future of this multispecies assemblage, through the introduction of a novel concept and practice.

This means that the concept and practice of ‘Siviri conservation’ as part of a multispecies assemblage is ontologically different from the concept of conservation used in Vanuatu national policy. The latter focuses on the preservation of either fish and shellfish as resources for humans or on maintaining biodiversity as a sphere separate from culture and society. Conservation in Siviri, however, is a world-making practice to preserve the local multispecies assemblage ‘Siviri marine management’ of which it is an important part for present and future generations.

## Footnotes

1 All citations of interlocutors in this article are translated from Bislama to English by the author.

2 While in the legal regulations of Vanuatu, the term ‘conservation area’ is mostly used as a designation for areas where it is prohibited to remove certain plants or animals, in some places in Vanuatu and in some parts of Oceania more widely, the expression ‘tabu area’ is common and sometimes both expressions are used by local actors. The term ‘marine protected area’ (MPA) is not prevalent in Vanuatu’s recent policy discourse (for a detailed explication of the various terms used in Vanuatu see Bartlett et al. 2010). In Polynesia, the name ‘rahui’ or ‘ra’ui’ is used for locally managed prohibitions to exploit marine areas.

3 Ni-Vanuatu is the self-designation of the people of Vanuatu.

4 The aim of this article is not to discuss whether ‘Siviri conservation’ is ‘conservation’ as defined by Western science (Smith and Wishie 2000).

5 I will not dwell on Edvard Hviding’s important case study on conservation in Marovo (Solomon Islands), where international NGOs are implementing projects for conservation because this is a quite different situation to Siviri. Important motivations for the Marovo people are to benefit from reciprocal relations with those organisations and to receive material goods, money and services from them (Hviding 2003, p. 551).

6 It is important to note that in this article I will deal with this marine conservation area only. This does not mean that Siviri villagers, depending on the context, also use ‘conservation’ to denote terrestrial conservation. Mostly, however, they refer to the marine conservation area, because Siviri does not (yet) have a terrestrial conservation area.

7 For a critical discussion of the current use of the concept ‘assemblage’ in recent works of cultural analysis see Marcus and Saka 2006.

8 I refer mainly to the work of scholars that can be attributed to the ‘recursive’ approach (see Salmond 2014a) as well as those who call their approach ‘political ontology’ (Blaser 2014).

9 I am aware of that the potentially different worlds I mention “are realities conjured by the presence (or absence) of things (entities, concepts, relations) encountered or generated within ethnographic relations” (Salmond 2014b, p. 168).

10 Paige West also describes the fundamentally different assumptions about the conservation of conservation professionals and that of the people participating in conservation activities – in her case the Maimafu Gimi in Papua New Guinea (West 2006, p. 44).

11 However, I am aware that “in seeking to distil alterity that arises ethnographically, we inevitably transform it into something other than itself” (Salmond 2017, p. 224).

12 Deleuze and Guattari in their example of a feudal assemblage included animals (horses) and things (stirrups) as well as statements, expressions and the juridical regime of heraldry etc. (Deleuze and Guattari 1987: 89).

13 For critical replies to advocates of the „ontological turn “ see, for example Bessire and Bond (2014), Carrithers (2010), Todd (2016) and Graeber (2015).

14 ‘Lokalisierung von globaler Klimawandelpolitik in Vanuatu: Rezeption von Wissen und kulturelle Transformationen’, funded by the Deutsche Forschungsgemeinschaft (DFG, German Research Foundation), grant number 298643416.

15 ‘A Sea of Connections: Contextualizing Fisheries in the South Pacific Region’ or SOCPacific (https://socpacific.net/) is co-funded by the Agence Nationale de la Recherche (grant number ANR-17-FRAL-0001–01) and the Deutsche Forschungsgemeinschaft (grant number 389654580). This work contributes to Future Earth Coasts, a Global Research Project of Future Earth.

16 This last period of fieldwork was conducted for the greater part as team research by me and Elodie Fache.

17 Bislama is a pidgin language and the lingua franca in Vanuatu.

18 For a more detailed history of community-based closures in Vanuatu see Bartlett et al. (2010).

19 In an amendment to this act in 2010, not only landowners but also customary resource stewards, recognised community leaders, village governing bodies, chiefs, family groups, tribes, organisations or bodies, private individuals and any other entity can apply for a community conservation area.

20 This plan was complemented, among others, by the Monitoring and Evaluation Framework in 2017.

21 This obviously relates to the Sustainable Development Goal No. 14 of the United Nations: “Conserve and sustainably use the oceans, seas and marine resources for sustainable development”.

22 An interview with one of the initiators of this network showed similarities regarding the motivations and values connected with conservation areas. A further explication of relations between the conservation areas of Siviri and Nguna/Pele goes beyond the intentions of this article.

23 For the role of Wan Smolbag for conservation efforts in Vanuatu see also Love (2021).

24 As the term ‘conservation area’ or, in Bislama. ‘konsevesen eria’ is very common in Siviri as well as on the national level, I will also use it (see also Bartlett et al. 2010, p. 101). During my research, working with women and men of the village community, I never heard them using the expression ‘tabu area’ when they spoke about Siviri’s conservation area, whereas some of my interlocutors explained that it is tabu (forbidden) to fish or collect shellfish in this area (Fig. 2).

25 Although quite common in other parts of Vanuatu (Bartlett et al. 2010: 100).

26 The ethnographic descriptions in this section are a mixed result of participant observation, interviewing and freelisting.

27 This does not mean, however, that tensions are totally absent.
